# Evaluation of the Surface Roughness of Bulk-Fill Composite Resins after Submission to Acidic and Abrasive Aggressions

**DOI:** 10.3390/biomedicines10051008

**Published:** 2022-04-27

**Authors:** Ionuț Tărăboanță, Dan Buhățel, Corina Alexandra Brînză Concită, Sorin Andrian, Irina Nica, Andra Claudia Tărăboanță-Gamen, Răzvan Brânzan, Simona Stoleriu

**Affiliations:** 1 Faculty of Dental Medicine, Grigore T. Popa University of Medicine and Pharmacy, 16 Universitatii Str., 700115 Iasi, Romania; ionut-taraboanta@umfiasi.ro (I.T.); sorin.andrian@umfiasi.ro (S.A.); irina.nica@umfiasi.ro (I.N.); andra-claudia.gamen@umfiasi.ro (A.C.T.-G.); razvan.branzan@umfiasi.ro (R.B.); simona.stoleriu@umfiasi.ro (S.S.); 2 Faculty of Dental Medicine, UMF Iuliu Haţieganu University of Medicine and Pharmacy, 4 Louis Pasteur Str., 400349 Cluj-Napoca, Romania

**Keywords:** bulk-fill composite resin, hydrochloric acid, surface roughness, toothbrush

## Abstract

This in vitro study aimed to assess the erosive effect of hydrochloric acid in association with toothbrushing procedure on the surface condition of three bulk-fill composite resins used for direct restoration. A total of 480 samples (160 from each composite resin): X-tra Fil (VOCO, Germany)—group A, Filtek Bulk-fill Posterior (3M-ESPE, St. Paul, MN, USA)—group B, G-aenial Posterior (GC Japan)—group C were prepared, submitted to chemical attack for 60 min with hydrochloric acid 30% and, subsequently, submitted to the abrasive effect of toothbrushing using 10,000 cycles with medium and hard bristles, at three different times (immediately and after 30 min after acid attack or without any chemical attack). The surface roughness of the samples was measured using a noncontact profilometer (Dektak XT, Bruker, Billerica, MA, USA). The values were analyzed using ANOVA and post hoc Bonferroni tests, with a *p* < 0.05. Chemical attack for 60 min associated with one year of toothbrushing with toothbrushes having medium or hard bristles increase the surface roughness of tested bulk-fill composite resins. No differences were recorded between toothbrushing with medium or firm bristles immediately or 30 min after acidic challenge for each of the three bulk-fill composite resins. Exposure to hydrochloric acid determines no effect on surface roughness of bulk-fill composite resins.

## 1. Introduction

Increasing patients request for esthetic restorations on both anterior and posterior teeth and the decision taken on Minamata agreement in 2013 [1] regarding a gradual reduction in the use of dental amalgam made the composite resins to become the mostly used and recommended materials for direct restorations [2]. Scientific research in the field of dental biomaterials has focused mostly on improving the aesthetic, physical, and mechanical properties of composite resins, thus meeting the requirements of dental clinicians [3]. A very important characteristic that resin-based restoration materials must meet is the resistance to wear [3,4], as they are in continuous contact with chemical, physical, or mechanical aggression factors [5]. Acidic foods or beverages, intrinsic acids in digestive pathologies such as gastroesophageal reflux disease, toothbrushing, or dental contacts are some of the factors incriminated in the degradation of composite resin restorations [3,5]. The alteration of these materials occurs at the interface between the organic matrix and fillers and has undesirable effects on their properties: increase of surface roughness, decrease of microhardness, color alteration, or marginal microleakage [5].

Bulk-fill composite resins have been developed with the goal to simplify direct restoration techniques [6]. They have the advantage of being light cured in layers of 4–5 mm, so they can be applied in a single-layer technique and they can reduce the clinical steps and time needed for direct restorations [6,7]. Therefore, the development of this type of composite resin counteracted the disadvantages of the multi-layer techniques, such as the incorporation of air bubbles, the linkage between the increments, modeling of each composite resin layer as well as a longer treatment time [8,9,10]. They also have the advantage of presenting a reduced polymerization shrinkage which leads to a reduced deflection of the cusps [2,11]. To increase the depth of polymerization, manufacturers have resorted to a number of methods, such as reduction of the filler content, increase of the filler size, and the addition of photo-initiators [9].

Gastroesophageal disorder can be one of the unfortunate consequences of modern lifestyle and it can have a direct impact on the teeth and the restorations in the oral cavity [12,13]. In gastroesophageal reflux disease the transport of hydrochloric acid (or gastric acid) in the oral cavity can lead to erosion of dental hard tissues. It can also affect the materials for direct restoration by degradation of the polymer matrix followed by loss of fillers, thus resulting in an increase of surface roughness and an impairment of the microhardness [12,14].

The most common oral hygiene habit is toothbrushing, but the association between toothbrushes and toothpaste can have an abrasive effect on resin-based materials, which can increase the surface roughness [6]. In addition, previous studies have shown that hydrochloric acid in gastroesophageal reflux disease and the toothbrushing procedure can act synergistically, causing advanced wear of direct restorative materials [6,15,16]. However, only few studies have evaluated the combined effect of intrinsic acids action and toothbrushing on bulk-fill composite resin.

This in vitro study aimed to investigate the effect of gastric acid and toothbrushing procedure on surface roughness of bulk-fill composite resins. The null hypothesis of this study is that gastric acid combined with toothbrushing do not increase the surface roughness of bulk-fill resins.

## 2. Materials and Methods

**Figure 1:**
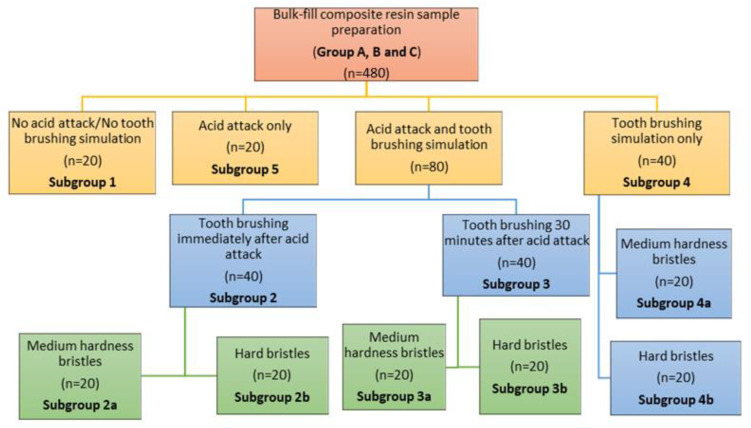
Design of the study.

The design of the study is represented in Figure 1. The detailed description of the materials used in the study is presented in Table 1.

### 2.1. Sample Preparation

Three different bulk fill composite resins: X-tra Fill (VOCO GmbH, Cuxhaven, Germany), Filtek Bulk-Fill (3M-ESPE, St. Paul, MN, USA), and G-aenial Posterior (GC Japan) were chosen for this study. The composition of the materials is presented in Table 1. A total number of 480 samples were prepared (160 samples from each material) and included in three groups (groups 1, 2, and 3), corresponding to each material used. The samples consisted in disks having 2 mm height and 6 mm diameter. They were obtained by inserting the material in an acrylic mold placed on a glass plate. Each material selected for the study was applied in the mold in one increment and was covered with another glass plate. A transparent matrix was applied at the interface between the material and the glass slabs in order to create a smooth surface. A constant pressure of 500 g was applied on the glass plate for 30 s, in order to remove the excess material and to avoid the air bubbles formation. The composite resin was then light-cured for 40 s through the glass plate using a LED light-curing lamp (Woodpecker LED.E, Guangxi, China) with a light intensity of 1000 mW/cm^2^, and a wavelength ranging from 420 to 480 nm. After removing from the mold the samples were submersed in distilled water for 24 h.

### 2.2. Finishing and Polishing Procedure

Sof-Lex system (Batch No. NC11342, 3M ESPE, St. Paul, MN, USA) was used to polish the samples. This system is composed by two wheels, beige and white. Both are of thermoplastic elastomer impregnated with aluminum oxide particles. The beige spiral was used for removing the scratches, smoothing, and finishing and the white one was used for final polishing. For each sample a beige spiral wheel and a white spiral wheel were used once for 1 min (30 s for each wheel). Both wheels were used at a speed of 20,000 revolutions per minute.

After this procedure 80 samples from each group were subjected to submersion in hydrochloric acid. In each group 20 samples were maintained as they resulted after finishing and polishing procedure (subgroup 1), 100 samples were exposed to hydrochloric acid action, and 40 samples followed a toothbrushing simulation process. The distribution of the samples in groups is detailed in Figure 1.

### 2.3. Simulation of Acid Attack

A total of 300 samples (100 from each group) were exposed to acidic challenge. The simulation of gastric acid attack was performed by using a 30% hydrochloric acid solution with a pH of 2.12. The pH value was verified with a portable pH-meter (Thermo Scientific Eutech pH 5+, Vernon Hills, IL, USA). The samples were submersed to acid challenge in a single cycle of 60 min, at a constant temperature of 37 °C in an incubator (Biobase BJPX-H30II, Biodusty, Shandong, China). After that the samples were submersed in distilled water at 37 °C, for 24 h. A total of 20 samples from each group were submersed in the acidic solution only (subgroup 5) and 80 were submitted to acid action followed by toothbrushing simulation process (subgroups 2 and 3).

### 2.4. Toothbrushing Simulation

Toothbrushing simulation was performed immediately after acid submersion for 40 samples from each group (subgroup 2) and 30 min after acid submersion for other 40 samples (subgroup 3). A total of 40 samples in the groups were exposed to toothbrushing procedure only (subgroup 4). For toothbrushing process simulation was used a brushing device having the frequency of 100 cycles/min, an intensity of 10,000 brushing cycles and the constant load of 200 g. For half of the samples in each subgroups 2, 3, and 4 toothbrushing simulation was performed using toothbrushes with medium bristle hardness (Colgate^®^ 360° Charcoal Toothbrush, Colgate-Palmolive Company, New York, NY, USA) (subgroups 2a, 3a, and 4a) and for the other half of the samples using toothbrushes with hard bristle hardness (Colgate^®^ Extra Clean Toothbrush, Colgate-Palmolive Company, New York, NY, USA) (subgroups 2b, 3b, and 4b). A toothpaste slurry composed of 1 g of a midrange abrasiveness toothpaste (Sensodyne, GSK, Middlesex, UK) and 1 mL of distilled water was used. After toothbrushing simulation, the samples were rinsed under running water for 2 min and then dried for 2 min using the air spray from the dental unit.

### 2.5. Surface Roughness Measurement

Profilometric evaluation was performed to analyze the surface characteristics of each sample using a non-contact profilometer Dektak XT (Bruker, Billerica, MA, USA). Bruker Software (Bruker, Billerica, MA, USA) was used to perform this action. Profilometric profiles were registered and arithmetic mean roughness values (Ra) was recorded. For each sample the mean Ra value was reported as a result of three determinations, each sample being rotated with a 90° angle.

### 2.6. Statistical Analysis

To compare the data between/within the groups and subgroups IBM SPSS 26.0 was used for statistical analysis of the values. Where significant differences were found, parametrical tests ANOVA and *post hoc* Bonferonni were used to identify specific differences between the surface roughness (Ra) values between/within study groups at a significance level of *p* = 0.05.

## 3. Results

**Figure 2:**
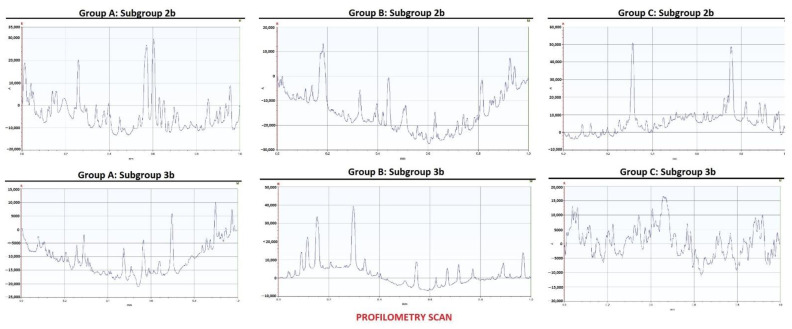
Profilometric measurements for two samples from groups A, B, and C in subgroups 2b and 3b.

Profilometric measurements of two samples from each group in subgroups 2a and 3a are presented as examples in Figure 2. The mean Ra values and standard deviation in each group and subgroup are presented in Table 2.

No statistically significant differences were recorded between groups A, B, and C in all subgroups at a significance level of 0.05 (Table 2).

In each study groups, significant differences were found between the subgroups at a significance level of 0.05 (Table 3). In group A, differences were recorded between subgroups: 1 and 2a (*p* = 0.016); 1 and 2b (*p* = 0.001); 1 and 3a (*p* = 0.001); 1 and 3b (*p* = 0.00); 1 and 4a (*p* = 0.001); 1 and 4b (*p* = 0.0001); 5 and 2b (*p* = 0.022); 5 and 3a (*p* = 0.029); 5 and 3b (*p* = 0.007); 5 and 4a (*p* = 0.019); and 5 and 4b (*p* = 0.013).

In group B, statistically significant differences were recorded between subgroups: 1 and 2a (*p* = 0.00001); 1 and 2b (*p* = 0.00); 1 and 3a (*p* = 0.00002); 1 and 3b (*p* = 0.00); 1 and 4a (*p* = 0.0001); 1 and 4b (*p* = 0.00); 5 and 2a (*p* = 0.01); 5 and 2b (*p* = 0.005); 5 and 3a (*p* = 0.004); 5 and 3b (*p* = 0.001); 5 and 4a (*p* = 0.027); and 5 and 4b (*p* = 0.001). 

In group C, statistically significant differences were recorded between subgroups: 1 and 2a (*p* = 0.00); 1 and 2b (*p* = 0.00); 1 and 3a (*p* = 0.00001); 1 and 3b (*p* = 0.00); 1 and 4a (*p* = 0.00001); 1 and 4b (*p* = 0.00); 5 and 2a (*p* = 0.006); 5 and 2b (*p* = 0.001); 5 and 3a (*p* = 0.0001); 5 and 3b (*p* = 0.00); 5 and 4a (*p* = 0.001); and 5 and 4b (*p* = 0.001).

## 4. Discussion

In the present study, the surface condition of bulk-fill composite resins was affected by the abrasive action of the toothbrush associated or not with the chemical attack of hydrochloric acid. Therefore, the null hypothesis was rejected. Increased surface roughness of resin-based restorations can influence mechanical strength, wear resistance, aesthetic properties, and the degree of bacterial biofilm accumulation [5,17]. According to a study conducted by Roche et al., saliva has the quality of neutralizing acids in the oral environment within 3 min of their appearance. Also, the concentration of acids decreases progressively as saliva exerts its buffering capacity [15]. For this reason in the present study we chose to use hydrochloric acid with a concentration of 30%. Bollen et al. reported the value of 0.2 µm as a critical value of surface roughness for bacterial adhesion [18]. Söderholm et al. reported that conditions in the oral cavity may influence the longevity of composite resin restorations [19]. Also, when comparing the behavior in the oral environment of several types of composite resins, they noticed that nanofill composites are more soluble compared to hybrid ones, based on the size of the filler particles [20]. In the present study, we did not find any differences between nanofilled and hybrid bulk-fill composite resins after exposure to acid attack with hydrochloric acid. The surface chemical degradation of resin-based materials caused by acid aggressions can be explained by the hydrolysis of ester radicals from bis-GMA, UDMA, TEGDMA monomers [21,22], and the subsequent formation of carboxylic acid and alcohol molecules which increase the degradation rate of the organic matrix [6,23]. The chemical modification of the polymer matrix will allow the exposure of the fillers and consequently will increase the surface roughness of the restoration [22,24,25]. The presence in the resin matrix composition of high molecular weight monomers such as Bis-GMA, UDMA, DDDMA, or AUDMA having fewer double bonds per unit can influence the viscosity, polymerization shrinkage and the aging degree of the material [22,26]. Although the three materials used in the study presented different ratio of organic matrix to filler, no significant differences were recorded. The results of our study are inconsistent with the findings of a study conducted by Wongkhantee et al. [27]. According to Sideridou et al. and El-Safty et al., no differences were found in the surface roughness of the composite resins based on different monomers (UDMA, Bis-GMA, and TEGDMA) after finishing and polishing procedure [28,29]. The finishing and polishing procedure was performed for 1 min (30 s for each wheel), according to manufacturer’s instructions.

Ghiorghe et al. reported that the coupling agent has a special importance in the wear resistance of composite resins by reducing the degradation of the filler through hydrolysis phenomena [22]. The water from the solutions can enter into the polymer structure and degrade it thus forming monomers and oligomers that are progressively released through the pores of the organic matrix [6,30]. The modification of the resin-based materials may be caused by the weak link between the organic and the inorganic components, due to the insufficient silanization of the fillers [31] or due to the hydrolytic degradation produced by the water sorption [6,31]. In addition, the increased water absorption can increase the osmotic pressure at the organic matrix/filler interface and, thus, can increase the surface roughness, followed by cracks appearance [6,32,33]. Although a number of studies have concluded that the wear resistance of resin-based materials is directly related to the volume of inorganic fillers [34,35], in our study this could not be demonstrated, all composites having the same behavior during acid submersion.

Chimello et al. observed that microfilled and hybrid composite resins are more resistant than nanofilled resins due to larger particles which are more difficult to be removed [3]. In our study, this could not be demonstrated as there were no differences between nanofill and hybrid composite resins regardless the aggressions to which they were subjected.

In this study chemical aggression was associated with abrasive effect of brushing which is the most common dental hygiene procedure. The simulation of the brushing procedure was performed using a special device that performs back and forth movements, having four toothbrush sites. The number of brushing cycles and the pressure applied to the brush can vary greatly from individual to individual. Therefore, for in vitro simulation of this procedure we used the methodology described by Sexon and Philips [36]. According to their protocol, an individual performs a number of 15 brushing cycles in one brushing session. In the condition of making two brushing sessions per day a total number of 10,000 brushing cycles per year will be obtained. The same number of brushing cycles was applied in our study. Toothbrushing was performed with a slurry resulting by mixture of a medium-abrasiveness Sensodyne Fresh Mint toothpaste (RDA value of 90) and distilled water.

Profilometric measurements revealed an increase in surface roughness at the end of the brushing procedure associated or not with exposure to hydrochloric acid. According to similar studies, the mechanisms that explain this behavior could be the wear of the polymer matrix, the exposure and loss of fillers due to the degradation of the bond between the organic and the inorganic components or the loss of filler particles due to cracks in the resin matrix. Therefore, toothbrushing and chemical wear with hydrochloric acid can act synergistically by changing the surface condition of the resin-based materials [6,37].

Fonseca et al. observed that composite resins containing low molecular weight monomers like TEGDMA, are more prone to chemical and mechanical degradation when comparing to composites containing high molecular weight monomers [38]. However, in our study we did not find significant differences between the VOCO X-tra fil composite containing TEGDMA monomers and TEGDMA-free materials, either after toothbrush procedure, acid submersion or after the association of toothbrushing with the chemical attack.

All three types of bulk-fill composite resins used in the study presented values of surface roughness higher than 0.2μm after finishing and polishing steps, after the exposure to toothbrushing and after the exposure to brushing associated with acid attack with hydrochloric acid. This might favor the adhesion and development of the bacterial biofilm and can subsequently lead to the failure of the restoration [6,39,40].

The limitations of this study are represented by the impossibility of an in vitro model to perfectly replicate the oral environment in terms of salivary flow, saliva composition, microorganisms development, enzymes activity, or thermal variation [9,41]. Even if the study samples were maintained in hydrochloric acid and distilled water, we did not take in consideration factors such as aging, thermocycling, or chewing cycles. Another limitation was the exposure of the samples for a limited period of time during the study to a constant acid attack without taking in consideration any dilution process, as it happens in the oral environment. Future in vivo studies are needed in order to validate the results of this study.

## 5. Conclusions

The results of this in vitro study show that chemical attack caused by 30% hydrochloric acid for 60 min in conjunction with one year of tooth brushing using toothbrushes having medium hardness or firm bristles increase the surface roughness of some bulk-fill composite resins. Toothbrushing with medium or firm bristles immediately or 30 min after the acidic challenge have the same effect on surface characteristics of all three tested bulk-fill composite resins. In the conditions of this study the exposure to hydrochloric acid has no effect on surface state of bulk-fill composite resins. Present study creates new perspectives on restorative treatment using bulk-fill composite resins in patients having gastroesophageal reflux disease.

## Figures and Tables

**Table 1:** Composition of the tested bulk-fill composite resins.

Name of Flowable Composite Resin	Manufacturer	Composite Type	Batch No.	Resin Composition	Filler wt%/vol%
**X-tra Fil** **(XTF)**	VOCO GmbH, Cuxhaven, Germany	Hybrid	2026242	Bis-GMA, TEGDMA, UDMA	86 wt%/70 vol%
**Filtek** **Bulk-fill Posterior** **Restorative**	3M-ESPE, St. Paul, MN, USA	Nanofill	N938942	AUDMA, UDMA, DDDMA, AFM	76.5 wt%/58.4 vol%
**G-aenial** **Posterior**	GC Japan	Hybrid	1806191	UDMA, Dimethacrylate comonomers	77 wt%/65 vol%

Bis-GMA—Bisphenol A diglycidyl ether methacrylate; TEGDMA—Triethylenglycol dimethacrylate; UDMA—Urethane dimethacrylate; DDDMA—1, 12-Dodecanediol dimethacrylate; AFM—addition-fragmentation monomers; and AUDMA—aromatic urethane dimethacrylate.

**Table 2:** Mean Ra value and standard deviation for groups A, B, and C in each study subgroup and statistical differences between groups A, B, and C in each study subgroup.

Subgroups	1			2a			2b			3a			3b			4a			4b			5		
Groups	A	B	C	A	B	C	A	B	C	A	B	C	A	B	C	A	B	C	A	B	C	A	B	C
**A**	0.32 ± 0.07	*	*	0.432 ± 0.055	*	*	0.464 ± 0.056	*	*	0.461 ± 0.039	*	*	0.475 ± 0.029	*	*	0.465 ± 0.029	*	*	0.469 ± 0.042	*	*	0.354 ± 0.037	*	*
**B**	*	0.329 ± 0.065	*	*	0.463 ± 0.021	*	*	0.468 ± 0.032	*	*	0.47 ± 0.025	*	*	0.478 ± 0.038	*	*	0.456 ± 0.013	*	*	0.483 ± 0.023	*	*	0.38 ± 0.016	*
**C**	*	*	0.309 ± 0.077	*	*	0.451 ± 0.045	*	*	0.466 ± 0.026	*	*	0.476 ± 0.016	*	*	0.489 ± 0.023	*	*	0.463 ± 0.015	*	*	0.484 ± 0.016	*	*	0.355 ± 0.023

* Not significant (*p* < 0.05).

**Table 3:** Statistical differences between study subgroups in groups A, B, and C.

	Group A								Group B								Group C							
	1	2a	2b	3a	3b	4a	4b	5	1	2a	2b	3a	3b	4a	4b	5	1	2a	2b	3a	3b	4a	4b	5
**1**	-	**	**	**	**	**	**	*	-	**	**	**	**	**	**	*	-	**	**	**	**	**	**	*
**2a**	**	-	*	*	*	*	*	*	**	-	*	*	*	*	*	**	**	-	*	*	*	*	*	**
**2b**	**	*	-	*	*	*	*	**	**	*	-	*	*	*	*	**	**	*	-	*	*	*	*	**
**3a**	**	*	*	-	*	*	*	**	**	*	*	-	*	*	*	**	**	*	*	-	*	*	*	**
**3b**	**	*	*	*	-	*	*	**	**	*	*	*	-	*	*	**	**	*	*	*	-	*	*	**
**4a**	**	*	*	*	*	-	*	**	**	*	*	*	*	-	*	**	**	*	*	*	*	-	*	**
**4b**	**	*	*	*	*		-	**	**	*	*	*	*	*	-	**	**	*	*	*	*	*	-	**
**5**	*	*	**	**	**	**	**	-	*	**	**	**	**	**	**	-	*	**	**	**	**	**	**	-

** Statistically significant (*p* < 0.05) * Not significant.

## Data Availability

Not applicable.
